# Computer-Guided Implant Surgery in Fresh Extraction Sockets and Immediate Loading of a Full Arch Restoration: A 2-Year Follow-Up Study of 14 Consecutively Treated Patients

**DOI:** 10.1155/2015/824127

**Published:** 2015-05-12

**Authors:** M. Daas, A. Assaf, K. Dada, J. Makzoumé

**Affiliations:** ^1^Department of Prosthodontics, René Descartes University, Paris, France; ^2^Private Practice, 62 Boulevard de la Tour Maubourg, 75007 Paris, France; ^3^Department of Prosthodontics, Beirut Arab University, Beirut, Lebanon; ^4^Department of Prosthodontics, Lebanese University, Beirut, Lebanon; ^5^Former Clinical Associate, Louis Mournier Hospital, Colombes, France; ^6^Department of Removable Prosthodontics, Saint-Joseph University, Beirut, Lebanon

## Abstract

*Statement of Problem*. Low scientific evidence is identified in the literature for combining implant placement in fresh extraction sockets with immediate function. Moreover, the few studies available on immediate implants in postextraction sites supporting immediate full-arch rehabilitation clearly lack comprehensive protocols. *Purpose*. The purpose of this study is to report outcomes of a comprehensive protocol using CAD-CAM technology for surgical planning and fabrication of a surgical template and to demonstrate that immediate function can be easily performed with immediate implants in postextraction sites supporting full-arch rehabilitation. *Material and Methods*. 14 subjects were consecutively rehabilitated (13 maxillae and 1 mandible) with 99 implants supporting full-arch fixed prostheses followed between 6 and 24 months (mean of 16 months). Outcome measures were prosthesis and implant success, biologic and prosthetic complications, pain, oedema evaluation, and radiographic marginal bone levels at surgery and then at 6, 12, 18, and 24 months. Data were analyzed with descriptive statistics. *Results*. The overall cumulative implant survival rate at mean follow-up time of 16 months was 97.97%. The average marginal bone loss was 0,9 mm. *Conclusions*. Within the limitations of this study, the results validate this treatment modality for full-arch rehabilitations with predictable outcomes and high survival rate after 2 years.

## 1. Introduction

For 40 years, the use of osseointegrated implants has shown to be a supplementary modality for treating full or partial edentulism [1]. Since the early 1990s, providing shorter treatment periods to patients has become a major focus first via the one-stage surgical technique [2] and then through the immediate loading protocol [3]. Delivering a fixed prosthesis on the same day of the last extractions supported by immediate implants has quickly become a major challenge. Patients can therefore never be left without teeth and the treatment length is ultimately shortened.

These protocols provide multiple benefits [4]: (1) only one surgical session, (2) immediate loading of a temporary prosthesis allowing for a reduction of the patient's discomfort and facilitating his return to social and professional life, (3) avoiding the resorption of hard tissues, the two-thirds of this reduction occurring during the first 3 months, (4) guiding the soft tissue healing for an optimal aesthetic environment and minimal recession, and (5) taking advantage of the extraction socket healing potential.

Nevertheless, if treatments by immediate implants associated with deferred loading have a long clinical history [5] and offer good results [6], low scientific evidence exists for their combination with immediate function.

The published clinical results are somewhat unsettled. Balshi and Wolfinger 1997 [7] and then Chaushu et al. 2001 [8] obtained success rates of 80% and 82.4%, respectively, for immediately loaded implants in postextraction sites. More encouraging results were reported by Cooper et al. 2002 [9] as well as by Grunder 2001 [10] with survival rates of 100% and 97.3%, respectively, for similar protocols in the mandible. However, it was reported by de Sanctis et al. 2009 [11] that in spite of achieving predictable osteointegration when implants were placed in fresh extraction sockets, the occurrence of buccal bone resorption may limit the use of this surgical approach.

More recently, Villa and Rangert 2005 [12] reported a 100% success rate for the treatment of 20 patients with 97 implants placed in postextraction sites and combined with early function. They demonstrated that, with an appropriate biomechanical, surgical, and medical protocol considering preservation of high implant stability and controlled inflammatory response, implants may be successfully osseointegrated when immediately placed and early-loaded in postextraction sites.

Moreover, the few studies available on immediate implants in postextraction sites supporting immediate full-arch rehabilitation are focused on the surgical part of the procedure and clearly lacked comprehensive prosthetic protocols whereas the NobelGuide concept (NobelBiocare AB) presents a step by step treatment procedure that is known to be meticulous and successful [13]. The purpose of this study is to evaluate the effectiveness of a protocol combining a computed tomographic scan-derived surgical template with an immediate implant placement in postextraction sites together with immediate temporization and loading.

## 2. Materials and Methods

In this prospective case series study, clinical and radiological data analysis were carried out over a two and half years period, on a total of 14 consecutively treated subjects (mean age 58.14 years) to be restored with fixed full arches prosthesis: 6 women and 8 men were treated via immediate implantation combined to CAD-CAM technology (NobelGuide, NobelBiocare AB).

### 2.1. Inclusion Criteria

The authors defined the following inclusion criteria in patient selection:

1. noncontributory medical history such as uncontrolled diabetes, and osteoporosis.
2. adequate bone volume and density for conventional dental implant placement as determined by CBCT without the need for bone or soft-tissue grafts,
3. patients requiring clearance of all remaining maxillary teeth,
4. no infected sockets.

### 2.2. Exclusion Criteria

Consider the following:

1. heavy smokers and/or confirmed bruxing subjects,
2. the total or partial lack of the above 4 inclusion criteria.

During preliminary evaluation, medical history and subjects' consent were collected. Preliminary screenings, including intraoral and panoramic radiographs, were performed. Eligible subjects received oral hygiene instructions. A total of 99 implants with external hexagon (NobelSpeedy and NobelBiocare AB) and oxidized surface (Ti-unite Groovy and NobelBiocare AB) were inserted and loaded immediately after surgery via previously manufactured lab-made prosthesis (Table 1). All surgeries were performed by one clinician and procedures were preplanned according to the collected data. Outcome measures were prosthesis and implant success, biologic and prosthetic complications, pain, oedema, and radiographic marginal bone levels evaluation at surgery and then at 6, 12, 18, and 24 months.

### 2.3. Protocol

**Figure 1:**
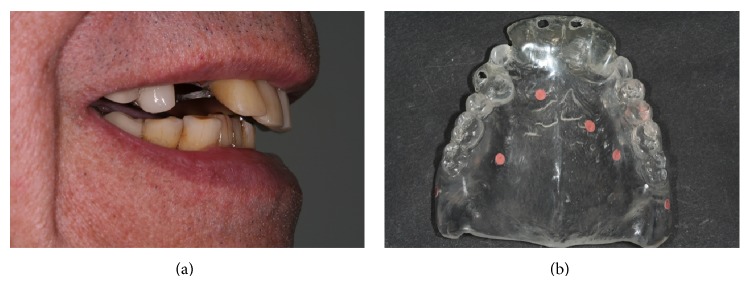
(a) Preoperative view demonstrating failing maxillary dentition. The treatment plan includes immediate implants in combination with CAD-CAM technology and their immediate loading with a prefabricated fixed prosthesis. (b) A radiographic template is realized according to the protocol for partially edentulous patient: verification windows to assess correct seating of radiographic template and radiopaque markers placed below gingival plane.

Impressions were made as for conventional partial removable denture, followed by intermaxillary relation registration. A trial denture was fabricated, tried in subject's mouth to validate the accuracy of the interarch relationship, and then processed to obtain a radiographic template according to the NobelGuide protocol (e.g., with at least 6 radiographic markers) (Figure 1). A first cone beam computed tomography (CBCT) was made with the radiographic template in place.

**Figure 2:**
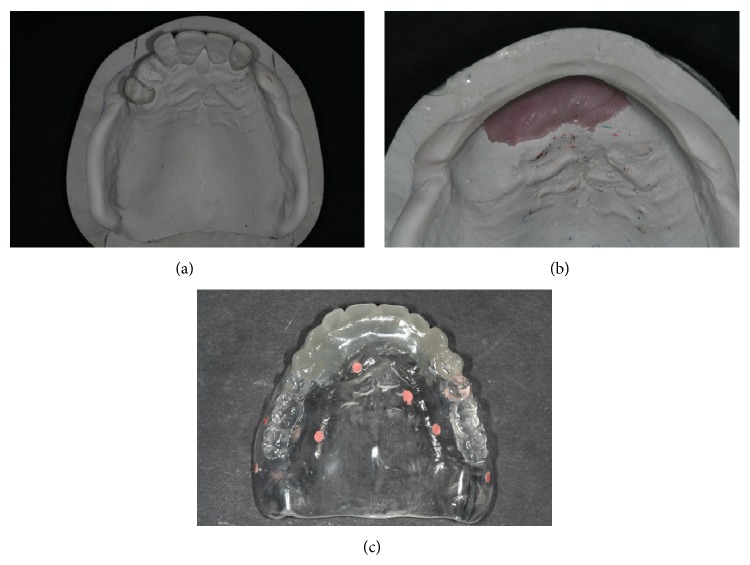
((a) and (b)) Teeth are cut off from the master model according to the periodontal probing data. In this case, an additional osteotomy has been planned according to the prosthetic needs. (c) The radiographic template is repositioned on the master model and an additional set-up is done. This additional set-up is then polymerized. Observe that the location of the radiographic markers at this stage must remain the same.

The remaining teeth were removed from the master model. Ridge (shape and volume) were regularized according to both the clinical findings and the CBCT findings (Figure 2). Two parameters were of particular consideration, the data collected during the periodontal examination and the prosthetic needs. The clinical findings included the probing depth, initial radiographic survey, and the preliminary planning following the first CBCT. In case of reduced prosthetic space, an additional osteotomy was performed but with caution, as resorption inevitably follows any surgical procedure. Still, a subcrestal leveling of the implants at the planning phase was programmed with implant platform positioned 2 mm under the coronal part of the vestibular alveolar crest.

Teeth that have been removed on the modified master model were replaced with denture teeth. The radiographic template was also altered so to be used for a second CBCT (e.g., scanning of the prosthesis itself) without any modification of the radiographic markers' position.

**Figure 3:**
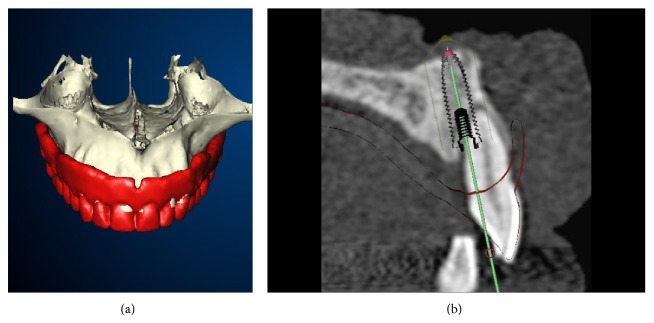
(a) Even if this template has never been worn by the patient, it can be merged to the patient CT data because of the radiographic markers. (b) Respect of the prosthetic project is easier with the computer-aided technology than in conventional hands-free procedure for immediate implants.

**Figure 4:**
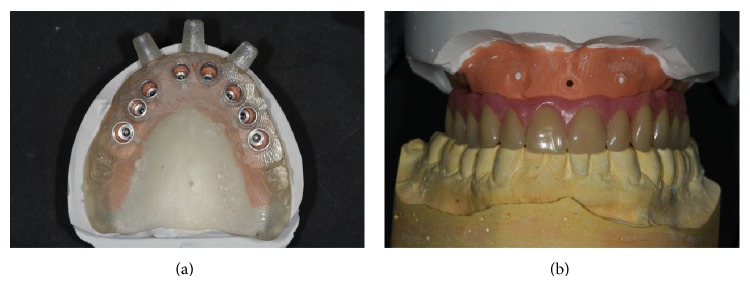
(a) Implant replicas are combined with the surgical template to obtain a master model. (b) The full-arch rehabilitation is realized on this model before the surgical session.

Finally, subject's data and prosthesis data were loaded into the Procera software (ProceraCadDesign, NobelBiocare AB) and a high resolution 3D model was then created. Planning was performed according to conventional protocols. However, it seemed to be more effective with this immediate implants procedure (Figure 3) for the following reasons: (1) implant's length and diameter were easier to choose to assure enough primary bone anchorage, (2) remaining bone areas could be used more effectively, and (3) implant positioning was made according to the prosthetic project which was perhaps the most difficult objective to fulfill in such procedures. Otherwise, the implant placement would be more dependent on the remaining bone volume than on the prosthetic project and the procedure a hands-free one. The surgical template was ordered and data were also used to prepare a master model that allows for the fabrication of an all-acrylic resin fixed prosthesis (Figure 4) before the surgical session.

### 2.4. Surgery

The surgical procedure was performed under local anesthesia with articaine chlorhydrate containing epinephrine 1 : 100,000 (Alpha spe, Dentsply). All subjects were sedated with diazepam (Valium 10 mg, Roche, Basel, Switzerland). Antibiotics amoxicillin 875 mg and clavulanic acid 125 mg (Augmentin 2 g, Glaxo Smith Kline) were given 1 hour prior to surgery and daily for 6 days thereafter. Corticoids (Solupred 60 mg, Sanofi Aventis) were administered daily from the day of surgery until 4 days postoperatively. Analgesics, 500 mg mefenamic acid (Ponstan Forte, Wilton ParkHouse, Wilton Place, Dublin 2, Ireland), were given on the day of surgery and postoperatively for the first 4 days if needed.

**Figure 5:**
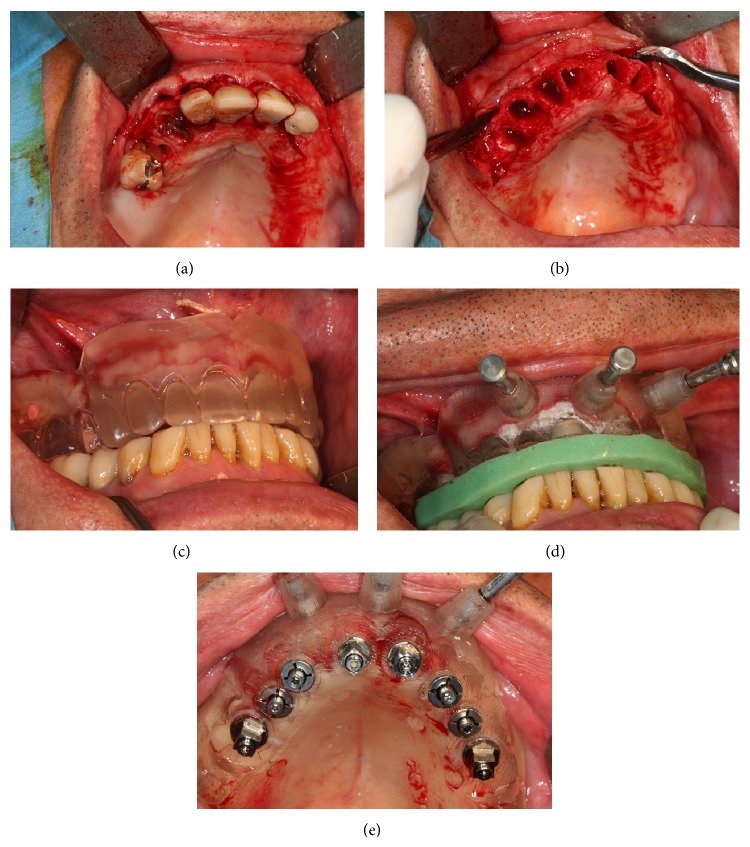
((a) and (b)) Extraction of the 6 remaining teeth. (c) Blanching of the mucosa in a homogeneous way proves the correct positioning of the conventional surgical guide. (d) The stereolithographic surgical guide can thus be correctly placed via a lab-made occlusal index (using the articulator) and then stabilized with 3 transfixation screws. (e) Eight implants have been inserted according to the NobelGuide procedure.

Three stages were followed in the procedure (Figure 5).

1. As with immediate complete denture cases, the remaining teeth were extracted, followed by osteotomy and/or soft tissue management when needed [14]. This regularization allowed for correct repositioning of the soft tissues close to the conditions of an edentulous ridge. It was reported from the master cast to the mouth via a transparent replica of the surgical stereolithographic guide (NobelGuide), fabricated in occlusion. Once made, these corrections secured an exact repositioning of the stereographic guide on the ridge.
2. Positioning of the surgical template in the correct interarch relationship. The difficulties of repositioning the vestibular flap were countered by a strict positioning of the surgical guide(s) in the correct interarch relation for a precise transfer of the surgical planning.
3. Implant placement per se. Implant length ranged from 7 to 13 mm.

### 2.5. Prosthetic Procedure

**Figure 6:**
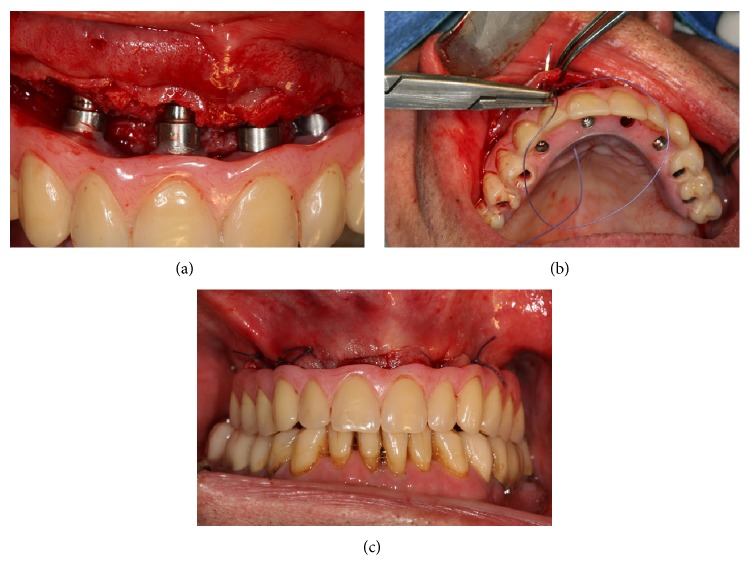
(a) Insertion of the restoration, in this protocol and because of the flap, the correct seating of the prosthesis can be checked visually. (b) The flap is sutured after the correct insertion of the restoration. (c) Final intraoral view.

Expandable abutments (Guided Abutments, NobelBiocare AB) were mounted in the provisional restoration. The bridge was then positioned over the implants and screw-retained by manual tightening. The correct abutment connection was checked visually (Figure 5) and assessed radiographically. The correct centric relation was verified and minor occlusal adjustments were performed when needed (Figure 6). The abutment screws were then tightened to 35 Ncm.

**Figure 7:**
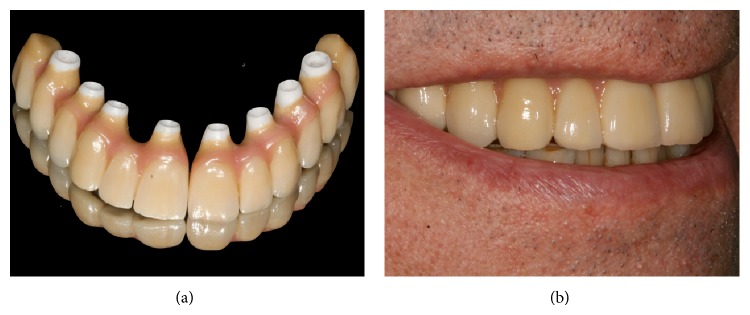
Final prosthesis. (a) Fixed ceramic one-piece prosthesis (Procera Implant Bridge Zirconia, Nobel Biocare AB). (b) Final extra oral view.

The subjects were enrolled in an implant maintenance program (Table 2) and a soft diet was instructed for 2 months. After 4 months, the prostheses were removed and the implants were individually tested for stability. If the implants were judged stable, the definitive fixed prosthesis was made as follows: two ceramic restorations (Procera Implant Bridge Zirconia, Nobel Biocare AB), seven metal-ceramic restorations, and fife hybrid prostheses (Figure 7).

### 2.6. Follow-Up

No subjects dropped out of this study. Subjects were examined at 1 week and at 1, 3, 6, and 12 months after the surgery. Examination included the assessment of prosthesis stability, peri-implant soft-tissue conditions, correct occlusion, and individual implant stability with the prosthesis removed at the 4-month follow-up.

To be classified as surviving, the implants were required to fulfill the following criteria: clinical stability, subject reported function without any discomfort, absence of suppuration, infection, or radiolucent areas around the implants.

**Figure 8:**
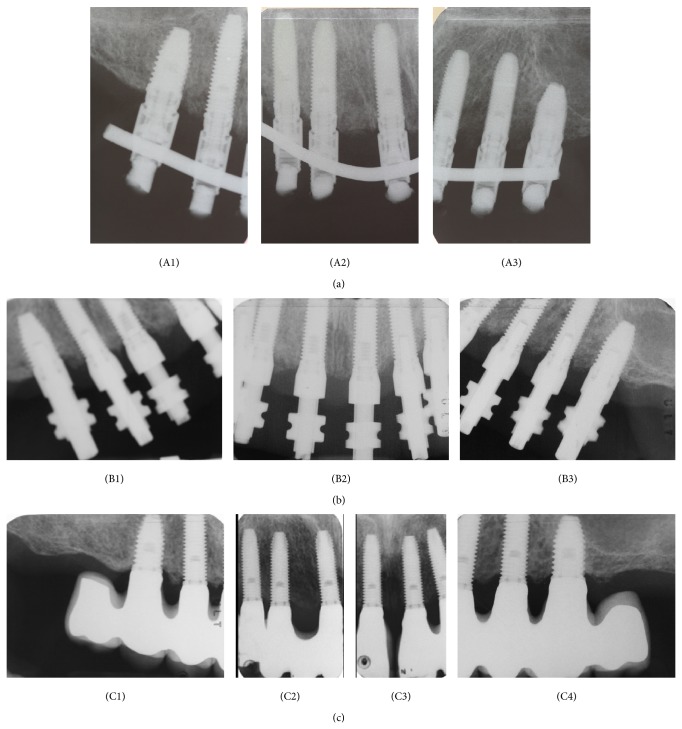
Radiographic examination: (A1), (A2), and (A3): postoperative radiographs. (B1), (B2), and (B3): at 6 months during the impression for the final restoration. (C1), (C2), (C3), and (C4): 18 months after the surgery.

Periapical radiographs (Figure 8) were made at implant insertion and then at 6-month intervals. The film was oriented with a conventional radiograph holder (Rinn XCP, Dentsply Rinn), manually positioned for an estimated orthogonal position of the film. An independent radiologist unaffiliated with the clinic interpreted the radiographs. The reference point for the reading was the implant platform. Marginal bone remodeling was calculated as the difference between readings at the examination and the baseline value at time of surgery. The radiographs were grouped as follows: implant insertion, 6 months, 1-year follow-up, 1-year and half follow-up, and 2-year follow-up. Implant survival and bone resorption data were analyzed with descriptive statistics.

## 3. Results

Implant survival rates are presented in Table 3. The cumulative survival rate at 2 years was 97.97%. Sixty six implants have passed the 1- to 2-year follow-up. The mean follow-up time was 16.15 months.

Two implants in two different subjects were lost after 4 months at time of substituting the provisional restorations with the permanent ones: one implant in the first molar position in one heavy bruxing subject and the other in the second premolar position and that already was not stable at the time of placement. Both implants were reinserted and were not included in the statistical analysis. The prosthesis in these two subjects survived with the support of the remaining implants.

Twelve subjects experienced slight postoperative pain and two subjects experienced moderate or severe pain. Slight oedema was recorded for 11 subjects and moderate or severe oedema for the remaining three.

Three subjects experienced fracture of the transitional acrylic resin prostheses. One of them practically did not follow the instructions of soft food diet in the first few months. The handling of these problems necessitated the repair of the prostheses, adjustment of occlusion, and night guard fabrication. No further mechanical complications occurred.

The radiographic assessment of marginal bone level concerning the 66 implants available at the end of the 2-year follow-up period showed a mean marginal bone loss of approximately 0,9 mm.

## 4. Discussion

Immediate implant placement in fresh extraction sockets has been investigated in several clinical studies, showing clear scientific evidence that osseointegration may be successfully achieved [11, 15]. Later, the growing need for avoiding temporary removable prostheses after surgery led to considering immediate loading of implants inserted in fresh extraction sockets, even in the chronically infected alveolar bone [16]. However, the few studies available on immediate implants in postextraction sites supporting immediate full-arch rehabilitation show lack of homogeneity and comprehensive protocols [17].

The number of studies investigating the clinical and radiological outcome of guided implant placement seems to confirm the high predictability of 3D planning software and indicate that immediate loading of oral implants yield acceptable to excellent results in full arch prosthetic restorations [14].

This preliminary study clearly demonstrates the high precision of transferring the virtual planning to the surgical field via the computer-aided technology even with extractions performed in the same surgical session. As in any extraction/implantation procedure, a judicious selection of the clinical case is essential for success. The presence of enough supra-alveolar bone is crucial for the primary stability of the fixture.

The benefits provided by computer-based planning seem to be superior with immediate implants cases in postextraction sites. Even without the use of the surgical template, there is a better match between the planned and the used implant when planning is done in a three-dimensional mode [18–20]. The preoperative choice of correct implant length and diameter can provide good primary stability through maximum filling of the extraction sockets and optimal engagement of the extra-apical alveolar bone. While no axial instability of any of the implants was observed, the insertion of some of the implants blocked at couples inferior to the recommended 35 Ncm. No adverse consequences were seen as these implants were connected to the others via a passive prosthesis. Knowing that primary stability measurements show significant correlations with different bone densities [21–23], the lesser density in the edentulous posterior regions could explain the encountered problems of stability, in opposition to the extraction sites where it was possible to engage in the nasal cortical bone.

Other benefits of guided implant planning and placement can be noted. (1) The remaining bone volume is used with more efficiency and predictability, (2) implant placement is made according to the prosthetic plan, and (3) the immediate cross-arch splinting of the freshly installed implant, another key factor for success [18, 19, 24–26], is easily obtained via the prefabricated prosthesis [27, 28].

The changes in marginal bone level were similar to those observed in the studies of Ganeles and Wismeijer in 2004 [24] and Glauser et al. in 2005 [25] on immediate loading and of Sanna et al. in 2007 on immediate loading and flapless surgery [26]. It can therefore be concluded that the applied protocol may improve the results of such prosthetic treatments renowned to be complex and unpredictable. It also offers a more adequate biomechanical environment for the implants, one that is “prosthetically driven.”

However, the successful use of this approach requires advanced clinical experience, surgical judgment, and proper case selection. Further studies with larger sample size, including control groups (full-arch immediate implant rehabilitations with delayed healing or with the absence of extraction sockets), are necessary to confirm the suggested protocol.

## 5. Conclusion

Within the limitations of this study, combining a computed tomographic scan-derived surgical template to an immediate implant placement in postextraction sites together with immediate temporization and loading seems to be a predictable therapy, with high survival rate at 2-year period and valid functional and aesthetic results when applied in selected cases.

The applied protocol provides a safer procedure for both surgeon and patient and may become the gold standard for such treatments.

More clinical trials and follow-up studies are necessary before final conclusions can be drawn in relation to the long-term safety and efficacy of this proposed protocol.

## Figures and Tables

**Table 1:** Quantitative data concerning size and number of the successful and failed implants used in 14 subjects.

Implant type	Length (mm)	Number	Failed
NobelSpeedy NP	13	4	0

NobelSpeedy RP	11.5	2	0
	13	55	1
	15	21	0

NobelSpeedy WP	7	1	0
	8.5	2	1
	10	7	0
	13	7	0

**Table 2:** Postsurgical maintenance protocol.

Maintenance protocol	
Day of surgery (day 1)	Panoramic and periapical radiographs, explanation of maintenance procedures to the patient, application of chlorhexidine gel after surgery, evaluation of occlusion, and instructions to avoid prosthesis overload.

Day 10	Application of chlorhexidine gel, control of suppuration by finger pressure, removal of the sutures, evaluation of occlusion, instructions to avoid prosthesis overload, and evaluation for fracture or loosening of prosthetic components.

Month 1	Application of chlorhexidine gel, control of suppuration by finger pressure, tightening of the guided abutments at 35 Ncm, evaluation of occlusion, instructions to avoid prosthesis overload, and evaluation for fracture or loosening of prosthetic components.

Month 4	Panoramic and periapical radiographs, removal of prosthesis for cleaning and disinfecting, application of chlorhexidine gel, evaluation for inflammation/infection, evaluation of occlusion, instructions to avoid prosthesis overload, and evaluation for fracture or loosening of prosthetic components.

Month 6 or at definitive prosthesis placement	Panoramic and periapical radiographs, oral hygiene procedures every 3 months without removal of the prosthesis, evaluation of occlusion, and evaluation for inflammation/infection.

Month 12 and after	Panoramic and periapical radiographs, oral hygiene procedures every 6 months without removal of the prosthesis, and evaluation of occlusion, evaluation for inflammation/infection.

Problem-related visit	Removal of prosthesis for disinfection and cleaning and for testing implants for infections and stability.

**Table 3:** Life table of cumulative survival rate for implants.

	Number	Failures	Withdrawn	CSR %
Placement to 6 months	99	2	0	97.97%
6 to 12 months	93	0	0	97.97%
1 to 2 years	66	0	0	97.97%

## References

[1] Brånemark P. I., Hansson B. O., Adell R. (1977). Osseointegrated implants in the treatment of the edentulous jaw. Experience from a 10-year period. *Scandinavian Journal of Plastic and Reconstructive Surgery*.

[2] Buser D., Weber H. P., Bragger U., Balsiger C. (1991). Tissue integration of one stage ITI implants: 3-year results of a longitudinal study with hollow-cylinder and hollow screw implants. *International Journal of Oral & Maxillofacial Implants*.

[3] Szmukler-Moncler S., Salama H., Reingewirtz Y., Dubruille J. H. (1998). Timing of loading and effect of micromotion on bone-dental implant interface: review of experimental literature. *Journal of Biomedical Materials Research*.

[4] Schropp L., Wenzel A., Kostopoulos L., Karring T. (2003). Bone healing and soft tissue contour changes following single-tooth extraction: a clinical and radiographic 12-month prospective study. *International Journal of Periodontics and Restorative Dentistry*.

[5] Anneroth G., Hedström K. G., Kjellman O., Köndell P.-Å., Nordenram A. (1985). Endosseus titanium implants in extraction sockets: an experimental study in monkeys. *International Journal of Oral Surgery*.

[6] Jo Y. H., Hobo P. K., Hobo S. (2001). Freestanding and multiunit immediate loading of the expandable implant: an up-to-40-month prospective survival study. *Journal of Prosthetic Dentistry*.

[7] Balshi T. J., Wolfinger G. J. (1997). Immediate loading of Brånemark implants in edentulous mandibles: a preliminary report. *Implant Dentistry*.

[8] Chaushu G., Chaushu S., Tzohar A., Dayan D. (2001). Immediate loading of single tooth implants: immediate versus non immediate implantation. A clinical report. *International Journal of Oral and Maxillofacial Implants*.

[9] Cooper L. F., Rahman A., Moriarty J., Chaffee N., Sacco D. (2002). Immediate mandibular rehabilitation with endosseous implants: simultaneous extraction, implant placement and loading. *International Journal of Oral and Maxillofacial Implants*.

[10] Grunder U. (2001). Immediate functional loading of immediate implants in edentulous arches: two year results. *International Journal of Periodontics and Restorative Dentistry*.

[11] de Sanctis M., Vignoletti F., Discepoli N., Zucchelli G., Sanz M. (2009). Immediate implants at fresh extraction sockets: bone healing in four different implant systems. *Journal of Clinical Periodontology*.

[12] Villa R., Rangert B. (2005). Early loading of interforaminal implants immediately installed after extraction of teeth presenting endodontic and periodontal lesions. *Clinical Implant Dentistry and Related Research*.

[13] Balshi S. F., Wolfinger G. J., Balshi T. J. (2006). Surgical planning and prosthesis construction using computed tomography, CAD/CAM technology, and the internet for immediate loading of dental implants. *Journal of Esthetic and Restorative Dentistry*.

[14] Meloni S. M., de Riu G., Pisano M., Tullio A. (2013). Full arch restoration with computer-assisted implant surgery and immediate loading in edentulous ridges with dental fresh extraction sockets. One year results of 10 consecutively treated patients: guided implant surgery and extraction sockets. *Journal of Maxillofacial and Oral Surgery*.

[15] Blus C., Szmukler-Moncler S., Khoury P., Orrù G. (2015). Immediate implants placed in infected and noninfected sites after atraumatic tooth extraction and placement with ultrasonic bone surgery. *Clinical Implant Dentistry and Related Research*.

[16] Villa R., Rangert B. (2007). Immediate and early function of implants placed in extraction sockets of maxillary infected teeth: a pilot study. *Journal of Prosthetic Dentistry*.

[17] Peñarrocha-Oltra D., Covani U., Peñarrocha-Diago M., Peñarrocha-Diago M. (2014). Immediate loading with fixed full-arch prostheses in the maxilla: review of the literature. *Medicina Oral, Patología Oral y Cirugía Bucal*.

[18] Jacobs R., Adriansens A., Verstreken K., Suetens P., van Steenberghe D. (1999). Predictability of a three-dimensional planning system for oral implant surgery. *Dentomaxillofacial Radiology*.

[19] Szmukler-Moncler S., Salama H., Reingewirtz Y., Dubruille J. H. (1998). Timing of loading and effect of micromotion on bone-dental implant interface: review of experimental literature. *Journal of Biomedical Materials Research*.

[20] Ozan O., Turkyilmaz I., Ersoy A. E., McGlumphy E. A., Rosenstiel S. F. (2009). Clinical accuracy of 3 different types of computed tomography-derived stereolithographic surgical guides in implant placement. *Journal of Oral and Maxillofacial Surgery*.

[21] Molly L. (2006). Bone density and primary stability in implant therapy. *Clinical Oral Implants Research*.

[22] Farré-Pagès N., Augé-Castro M. L., Alaejos-Algarra F., Mareque-Bueno J., Ferrés-Padró E., Hernández-Alfaro F. (2011). Relation between bone density and primary implant stability. *Medicina Oral, Patologia Oral y Cirugia Bucal*.

[23] Yoon H. G., Heo S. J., Koak J. Y., Kim S. K., Lee S. Y. (2011). Effect of bone quality and implant surgical technique on implant stability quotient (ISQ) value. *Journal of Advanced Prosthodontics*.

[24] Ganeles J., Wismeijer D. (2004). Early and immediately restored and loaded dental implants for single-tooth and partial-arch applications. *International Journal of Oral and Maxillofacial Implants*.

[25] Glauser R., Ruhstaller P., Windisch S. (2005). Immediate occlusal loading of Brånemark System TiUnite implants placed predominantly in soft bone: 4-Year results of a prospective clinical study. *Clinical Implant Dentistry and Related Research*.

[26] Sanna A. M., Molly L., van Steenberghe D. (2007). Immediately loaded CAD-CAM manufactured fixed complete dentures using flapless implant placement procedures: a cohort study of consecutive patients. *Journal of Prosthetic Dentistry*.

[27] Crespi R., Capparè P., Gherlone E., Romanes G. E. (2007). Immediate occlusal loading of implants placed in fresh sockets after tooth extraction. *International Journal of Oral and Maxillofacial Implants*.

[28] Gallucci G. O., Morton D., Weber H.-P. (2009). Loading protocols for dental implants in edentulous patients. *The International Journal of Oral & Maxillofacial Implants*.

